# Characterization of the Function of Two S1Fa-Like Family Genes From *Populus trichocarpa*

**DOI:** 10.3389/fpls.2021.753099

**Published:** 2021-10-04

**Authors:** Huimin Zhao, Yani Niu, Hao Dong, Yaqi Jia, Yucheng Wang

**Affiliations:** State Key Laboratory of Tree Genetics and Breeding, Northeast Forestry University, Harbin, China

**Keywords:** *Populus trichocarpa*, gene expression, S1Fa-like, drought stress, drought tolerance, transcription factor

## Abstract

S1Fa-like transcription factors (TFs) are small molecular weight proteins that contain both nuclear localization and DNA binding domains. However, the functions of S1Fa-like TFs are poorly understood. In the present study, we identified the S1Fa-like TFs from the *Populus trichocarpa* genome, which revealed two S1Fa-like TF genes, *PtS1Fa1* and *PtS1Fa2*. *PtS1Fa1* and *PtS1Fa2* expression was suppressed by drought and salt stress, and was also significantly altered by ABA, MeJA, or SA treatment. Both PtS1Fa1 and PtS1Fa2 are nuclear proteins. Transgenic *P. trichocarpa* plants overexpressing *PtS1Fa1* and *PtS1Fa2*, respectively, were generated. The plants overexpressing *PtS1Fa2* showed increased fresh weight, chlorophyll content, and root length and weight compared with those in wild-type (WT) *P. trichocarpa* under drought conditions. Meanwhile, these phenotype traits of plants overexpressing *PtS1Fa1* were similar to those of WT plants. Furthermore, overexpression of *PtS1Fa2* reduced the malondialdehyde (MDA) content, electrolyte leakage, H_2_O_2_ and O_2_- contents, and increased superoxide dismutase (SOD) and peroxidase (POD) activities. The expression of *SOD* and *POD* was also induced by *PtS1Fa2*. However, overexpression of *PtS1Fa1* failed to affect any of these physiological parameters or *SOD* and *POD* gene expression. These results suggested that *PtS1Fa2* plays a role in drought tolerance, and confers drought tolerance by increase antioxidant activity to reduce reactive oxygen species (ROS) accumulation.

## Introduction

As sessile organisms, plants have to cope with various abiotic stresses, such as low or high temperatures, drought, and salinity (Zhu, 2016). These abiotic stresses are major environmental factors that not only limit plant yield, but also affect their geographical distribution. For plants, adverse environmental conditions, including salt, drought, heavy metal, extreme temperatures, chemical toxicity, and nutrient deficiency, usually cause a series of physiological, morphological, molecular, and biochemical changes that negatively affect plant development, yield, growth, and survival worldwide (Huang et al., 2012; Sharma et al., 2013). To adapt to abiotic stress conditions, plants employ a series of cellular responses and biological pathways, such as enhanced antioxidants, the accumulation of compatible solutes, production of stress proteins as molecular chaperones, and salt overly sensitive (SOS) responses to adverse stress (Wang et al., 2003; Kang et al., 2009).

Transcription factors (TFs) function in regulation of gene expression, and are important gene expression regulators. Among the different TFs, S1Fa-like proteins comprise a family of a small molecular weight TFs, being small peptides (70 aa) with both nuclear localization and DNA binding domains. The expression of S1Fa-like was mainly expressed in roots and etiolated seedlings rather than in green leaves (Zhou et al., 1995). Interestingly, the number of S1Fa-like family TFs in different plant species vary highly. Most plant species contain no more than five S1Fa-like family TFs; however, some plant species have more than 100 S1Fa-like TFs. For instance, the genome of *Arachis hypogaea* contains 126 S1Fa-like TFs (Chen et al., 2016). The S1Fa-like TFs had been identified as membrane-bound transcription factors (MTF), which are usually stored in a dormant form, become active after being released from the membrane, and then transferred into the nucleus to perform its function (Che et al., 2010; Yao et al., 2017). In addition, the S1Fa-like gene is a key partner in cotton in response to abiotic stress. S1Fa-like TFs were one of the most abundant groups and most of the S1Fa-like family members were down-regulated by abiotic stress (Tahmasebi et al., 2019). S1Fa-like TFs were found in the leaves of *Brassica napus* after salt stress for 1 h, suggesting that they may regulate salt-responsive target genes (Yong et al., 2014). However, their function is mostly unknown (Chen et al., 2016), and have been poorly investigated until now (Gazara et al., 2019).

*Populus trichocarpa* Torr & Gray was the first woody plant whose genome was sequenced (Tuskan et al., 2006). *P. trichocarpa* has been considered as a model woody plant in the study of wood growth and properties, development, and biotic or abiotic stress responses. However, to date, there is still no report on functional characterization of *Populus* S1Fa-like TFs. In the present study, we searched for all members of the *Populus* S1Fa-like family in the genome of *P. trichocarpa*, and identified two S1Fa-like TF genes, *PtS1Fa1* and *PtS1Fa2*. We further investigated the expression of these two genes in response to abiotic stress. Then, transgenic *P. trichocarpa* plants overexpressing *PtS1Fa1* and *PtS1Fa2*, respectively, were generated, and their functions in drought stress were characterized. This study laid the foundation for further determination of the biological functions of the S1Fa-like family in *P. trichocarpa.*

## Materials and Methods

### Plant Materials and Growth Conditions

The plantlets of *Populus trichocarpa* were grown in the greenhouse at 23°C under a 16/8 h light/dark cycle and well-watered. To analyze the expression of S1Fa-like family genes in different tissues, the 5-weeks-old plantlets grown in soil was used for harvesting their tissues: including roots, mature stems, young stems, young leaves, and mature leaves. To assay the expression profiles of the genes for S1Fa-like family responding to drought, salinity stress or plant hormone, 5-weeks-old Populus plants grown in soil and well-watered plants were watered with a solution of 20% polyethylene glycol (PEG)6,000 or 150 mM NaCl at the roots. The plants were watered on their roots with the solution of 100 μM MeJA, 200 μM ABA or 100 μM SA, respectively, for 0, 1, 3, 6, 9, and 12 h. Each sample contains 3 plantlets. The samples were harvested and frozen in liquid nitrogen quickly, and were stored at −80°C for RNA isolation.

### Sequence Alignment and Phylogenetic Analysis of S1Fa Genes

To investigate the evolutionary relationship of the *S1Fa* gene family in *Populus*, S1Fa amino acid sequences were retrieved from *Populus euphratica*, *Arabidopsis thaliana*, and *Populus trichocarpa*. Amino acid sequence alignments of S1Fa proteins were performed using the program of MEGA (MEGA7) described by Tamura et al. (2011). The neighbor-joining (NJ) program of MEGA7 was used in construction of a phylogenetic tree.

### Gene Structure and Conserved Motif Analyses

The MEME program^1^ was employed for analysis of the conserved motifs among the studied genes with the parameters of a maximum motif number of 50. The Gene structure Display Server Program was used to determine the *S1Fa* gene member structure (Hu et al., 2015).

### Analysis of Chromosome Locations and Promoters of S1Fa Genes

The gff3 annotation files of the *P. trichocarpa* was used to retrieve the sizes of *P. trichocarpa* chromosome and the location of the *S1Fa* genes, and Mapchart v2.2 software was used to visualize the position of the *S1Fa* genes in the genome of *P. trichocarpa* (Wageningen University and Research, AA, Wageningen). The promoter sequences of the *S1Fa* genes (2,000-bp upstream of the start codon ATG) were retrieved from the *P. trichocarpa* genome using TB tools software (Chen et al., 2020). New PLACE^2^ was used to analyze the *cis-*acting elements of the *S1Fa* genes. The gene promoters containing S1F binding sites were screened by retrieving the *P. trichocarpa* genome to determine the number and location information of the S1F binding sites, and were visualized with the TB tool software.

### Generation of Transgenic *Populus trichocarpa* Overexpressing PtrS1Fa1 and PtrS1Fa2

To construct the overexpression vectors, the coding sequences (CDSs) of *PtrS1Fa1* and *PtrS1Fa2* were cloned separately into vector pROK2 under the control of cauliflower mosaic virus (CaMV) 35S promoter to generate the vectors p35S:PtrS1Fa1 and p35S:PtrS1Fa2. These vectors were introduced into *Agrobacterium tumefaciens* strain EHA105. Transformation of *P. trichocarpa* was then carried out according to the Agrobacterium infiltration method of Li et al. (2017) with minor modifications. In brief, stem explants (1.0 cm in length) were incubated in a solution containing Agrobacterium cells at OD600 of 0.5) for 20 min. Infected explants were co-cultured in the dark for 48 h, and moved to medium (supplemented with 50 mg/L kanamycin and 300 mg/L Cefotaxime) for bud induction. The kanamycin resistant shoots were transferred to rooting medium (supplemented with 50 mg/L kanamycin and 300 mg/L Cefotaxime) for root generation. All the sequences of primers were included in Supplementary Table 1.

### Treatments of Abiotic Stress

*S1Fa* transgenic and wild-type (WT) plant were grown in the greenhouse at 23°C under a 16/8 h light/dark cycle and well-watered. The plants were well watered for 5 weeks in the soil, and then were treated with 20% PEG6000 for 15 days. The phenotype was observed, and the plant height, fresh weight, root weight, root length and physiological were measured. The plants under normal conditions were used as the controls. Each sample contains 3 plantlets.

### The Subcellular Location of PtrS1Fa1 and PtrS1Fa2

The 3′ ends of the CDSs of *PtrS1Fa1* and *PtrS1Fa2*, without the stop codon, were fused separately with the 5′ end of the *GFP* gene controlled by 35S CAMV promoter to construct 35S: *PtrS1Fa1*-GFP and 35S: *PtrS1Fa2*-GFP vectors. The *GFP* gene driven by 35S CaMV promoter (35S: GFP) was employed as the control. All the constructs were transiently transformed into tobacco plants (*Nicotiana benthamiana*) using the method of *Agrobacterium* infiltration. After 72 h of incubation in the dark, the epidermal cells of the transformed tobacco were stained with 4′,6-diamidino-2-phenylindole (DAPI) (100 ng/mL) and visualized under an LSM800 confocal laser scanning microscope (Zeiss, Jena, Germany).

### Determination of Physiological Changes

The well rinsed samples were incubated in a vacuum for 15 min, and the electrical conductivity was detected as S1, followed by heating in a boiling water bath for 20 min after which the final electrical conductivity (S2) was measured. Electrolyte leakage was calculated as (S1/S2) × 100% (Lutts et al., 1996). The chlorophyll content was measured at 663 and 645 nm using a spectrophotometer. The procedures for staining of nitroblue tetrazolium (NBT) and 3′-diaminobenzidine (DAB) were according to the method of Zhang et al. (2011). In brief, the leaves were soaked with NBT (1 mg/mL NBT in 10 mM phosphate buffer with 10 mM sodium azide, pH 7.8) or DAB solutions (1 mg/mL DAB–HCl, pH 3.8). After 20% PEG6000 treatment for 9 h, and the detached leaves were used for DAB and NBT staining. Malondialdehyde (MDA) measurement was based on the color reaction produced by the reaction of MDA and thiobarbituric acid, according to Madhava Rao and Sresty (2000). The activities of superoxide dismutase (SOD) and peroxidase (POD) were determined following the procedure of Han et al. (2007). H_2_O_2_ levels were determined using the titanium tetrachloride precipitation method, according to Brennan and Frenkel (1977).

### RNA Extraction and Quantitative Real-Time Reverse Transcription PCR

According to the manufacturer’s instructions, the cetyltrimethylammonium bromide (CTAB) method (Chang et al., 1993) was used to extract total RNA from *P. trichocarpa*. ReverTra Ace^®^ qPCR RT Master Mix (Toyobo, Osaka, Japan) was used to reverse transcribe total RNA (about 1.0 μg) into cDNA with the volume of 10 μL. The reverse transcription product was diluted into 100 μL and used for Real-Time Reverse Transcription PCR (qRT-PCR). The reaction system for qRT-PCR contains 10 μl of SYBR Premix Ex Taq^TM^ (Toyobo), 1 μl of cDNA template, and the forward or reverse primers (each 1 μM). The thermal cycles were set as the following: 94°C for 30 s; then 40 cycles of 15 s at 94°C, 30 s at 57°C, and 45 s at 72°C on a qTower 2.2 real-time PCR system (Analytik Jena AG, Jena, Germany). The gene for *PtrActin2* was served as the internal control. The gene expression was determined according to the method of 2^–ΔΔ^CT (Livak and Schmittgen, 2001). All the primer sequences and GenBank numbers are included in Supplementary Table 1.

### Statistical Analysis

The Statistical Package for the Social Sciences (SPSS 22, IBM Corp., Armonk, NY, United States) was used for statistical analyses, and the data was calculated using One-way analysis of variance (ANOVA). The *P* value <0.05 was considered as statistical significance.

## Results

### Phylogenetic Study and S1Fa Genes Classification

**FIGURE 1:**
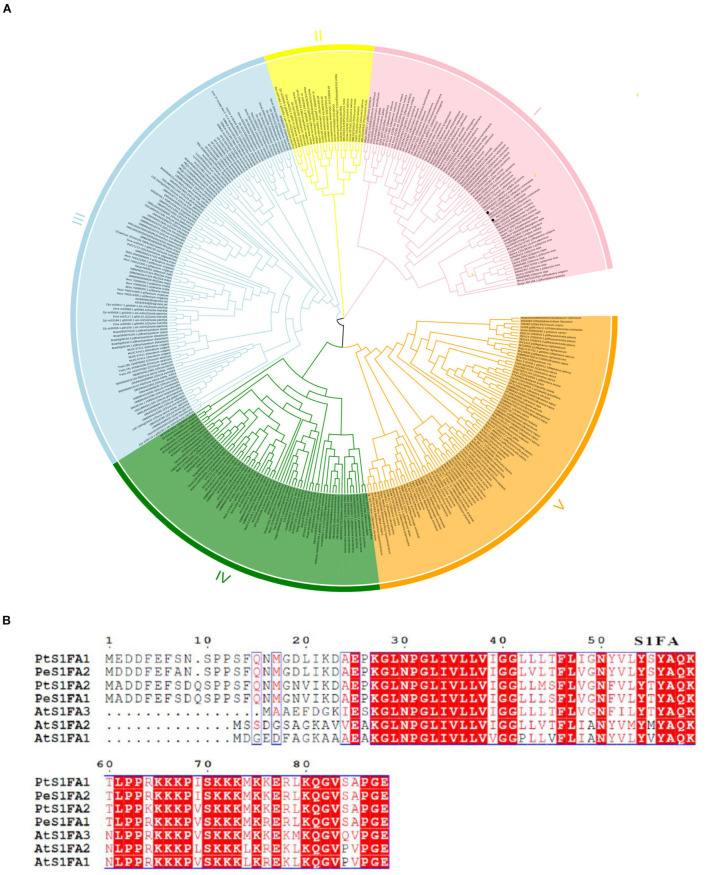
Phylogenetic analysis and multiple sequence alignment of S1Fa-like proteins. **(A)** Dendrogram of the protein sequences of the S1Fa-like gene family from some species with known genome. The dendrogram was constructed using MEGA version 7 software and using the method of neighbor-joining (NJ). Each special class is present by one color. **(B)** Multiple sequence alignment of S1Fa proteins from *Populus trichocarpa*, *Arabidopsis*, and *P. euphratica*. Multiple sequence alignment was performed using MEGA version 7 software. The amino acid with red background were highly conserved, and amino acid with red were conserved.

Two *S1Fa* genes were identified from the genome database of *P. trichocarpa*. The CDSs of the two *S1Fa* genes comprised 267 and 270 bp, encoding deduced proteins of 88 and 89 amino acids, respectively. To explore the relationships among S1Fa-like proteins, we used the amino acid sequences from all species with known genomes to constructed the NJ-phylogenetic tree. The phylogenetic tree suggested that these S1Fa-like proteins could be classified into five groups, suggesting that these S1Fa proteins in different group may have different functions. Among these 5 groups, group III has more members than other groups, including 108 members, and group II has the least quantity with 24 members. Group 1, 4, and 5 contains 75, 67, and 85 members, respectively. Both PtS1Fa1 and PtS1Fa2 belong to group V, which can be divided into 2 sub-groups. Both PtS1Fa1 and PtS1Fa2 are highly homologous to members of the *P. euphratica* S1Fa-like family (Figure 1A).

Multiple sequence alignments shows that the amino acid sequences of the S1Fa-like genes from *P. trichocarpa*, *Arabidopsis*, and *P. euphratica* are highly conserved, especially in their domains. The conserved domain of S1Fa was highlighted by the box, and the red background represents the identical amino acid sequence. PtS1Fa1 shares 95.4% sequence similarity with PeS1Fa2 from *P. euphratica*, and 72% sequence similarity with AtS1Fa1 from *Arabidopsis*. PtS1Fa2 shares 96.5% sequence similarity with PeS1Fa1 from *P. euphratica* (Figure 1B).

### Gene Structure and Conserved Motif of S1Fa Genes

**FIGURE 2:**
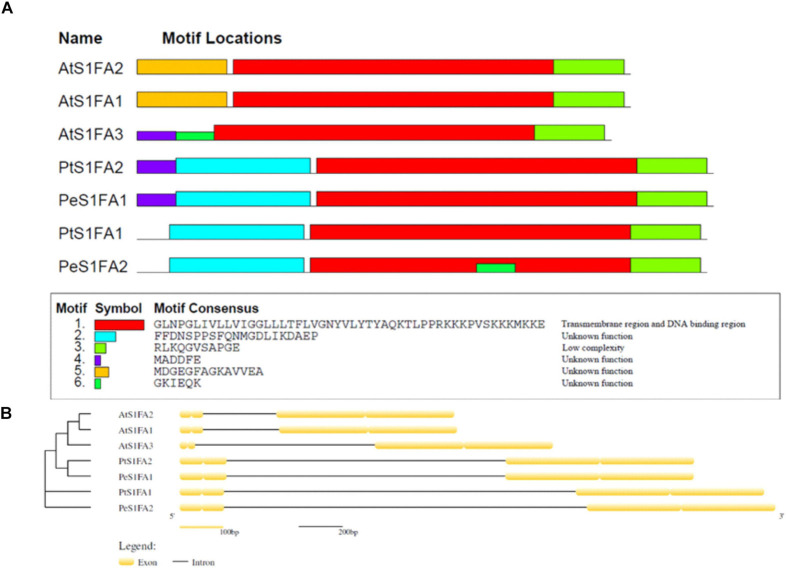
The protein motifs and gene structures of S1Fa-like gene family from poplar. **(A)** The protein motifs of the S1Fa-like family members. Different motifs were sketched with different colorful box. Clustering was carried out based on the phylogenetic results. **(B)** Gene structures. Exons and introns are displayed using yellow boxes and Black solid lines, respectively.

To investigate the features of *S1Fa*, conserved motifs of *S1Fa* genes from *P. trichocarpa*, *A. thaliana*, and *P. euphratica* were identified using the MEME database. Six conserved protein motifs in *S1Fa* members were analyzed. Smaller differences in the number of motifs (Figure 2A). All the studied *S1Fa* genes contain paramount motif 1 and motif 3. The *S1Fa* genes of *P. trichocarpa* and *P. euphratica* contain a common motif 2, and *S1Fa* genes from *Arabidopsis* contain unique motif 5. The two *S1Fa* genes from *P. trichocarpa* shared similar conserved motifs with the genes from *P. euphratica*, suggesting that they are homologous genes. The intron–exon distribution was analyzed to understand the structure of the *S1Fa* genes. Figure 2B shows the structure of exon–intron of the *S1Fa* genes. The structures of *S1Fa* genes from *P. trichocarpa*, *A. thaliana*, and *P. euphratica* are also highly similar. The numbers of introns and exons are identical among different groups, including 1 intron and 4 exons. Moreover, the introns are all located after the 2 exons at the nitrogen terminal. *PtS1Fa1* and *PtS1Fa2* shared highly similar gene structures and conserved motifs.

### Chromosome Distribution and *Cis-*Elements of Promoters Analysis

**FIGURE 3:**
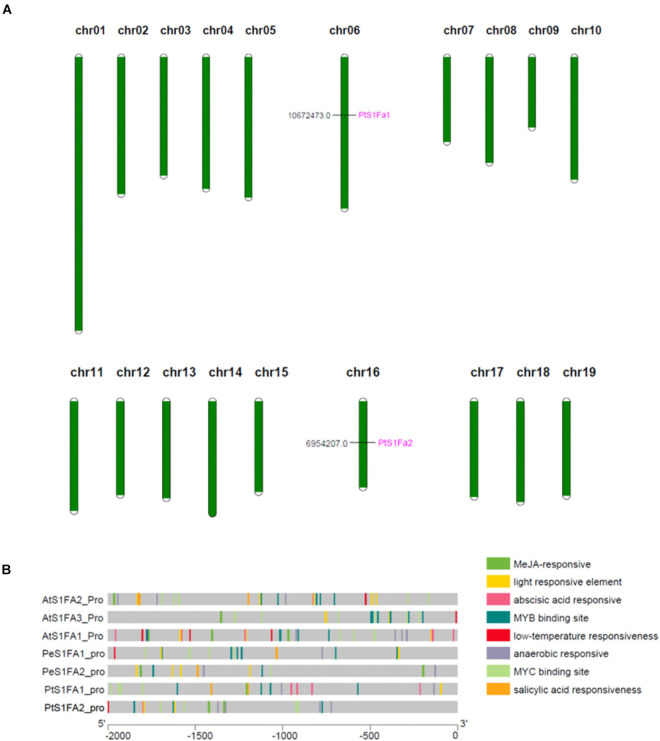
Chromosomal localization of the S1Fa-like genes and gene duplication events. **(A)** The tandem duplication events and distribution of chromosome of poplar S1Fa-like genes. Chr01–Chr19 indicated the number of chromosomes 01–19. **(B)** Prediction of the *cis-*elements in the S1Fa-like gene promoters. On the left are gene names according to the phylogenetic tree. Different patterned colors on the right represent different *cis-*elements.

According to genome annotation analysis, *PtS1Fa1* is located on chromosome 06 and *PtS1Fa2* is located on chromosome 16 (Figure 3A). Therefore, we identified the *cis-*acting elements in the promoters (2,000bp upstream of the translation start site) of *PtS1Fa1* and *PtS1Fa2* using Plant CARE online software (Figure 3B). In addition, we downloaded the 2,000bp sequences of the *S1Fa* gene sequences from *P. trichocarpa*, *A. thaliana*, and *P. euphratica*, and analyzed their a *cis-*acting element. The promoters of *S1Fa* genes contain a lot of MYB and MYC binding sites. In addition, there are a lot of *cis-*elements that respond to plant hormones, such as MeJA-responsive, salicylic acid (SA) responsiveness, anaerobic responsive, and abscisic acid (ABA) responsive elements. Promoter analysis showed that the *PtS1Fa1* promoter contains four MYB binding sites (MBS) that are related to drought response induction, four MYC binding sites, and four elements related to ABA responsiveness. The *PtS1Fa2* promoter contains three MBS related to drought response induction, four MYC binding sites, and four elements related to MeJA responsiveness (Figure 3B). Therefore, we suspected that *PtS1Fa1* and *PtS1Fa2* might be involved in plants’ response to abiotic stresses.

### Expression Pattern of the S1Fa-Like Gene Family in Poplar

**FIGURE 4:**
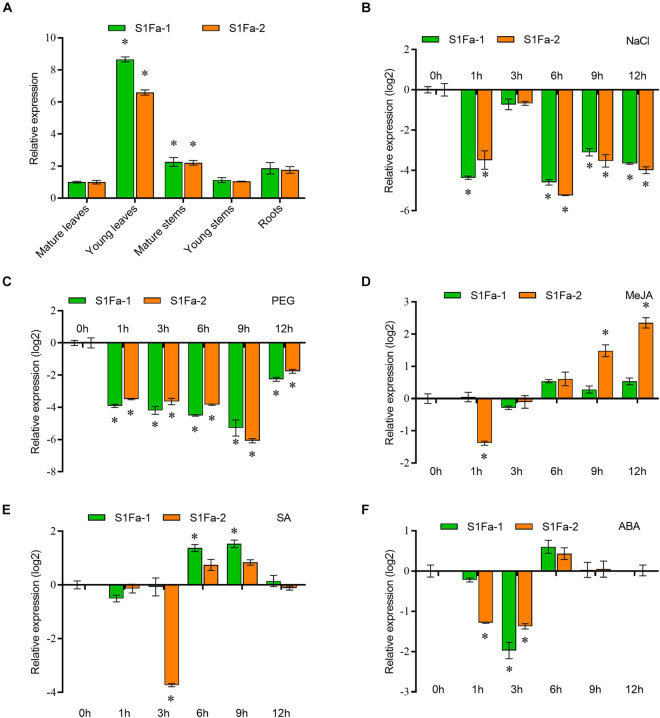
The expression patterns of *S1Fa-like* genes. **(A)** Expression of *PtS1Fa1* and *PtS1Fa2* in different tissues of *Populus trichocarpa*. Tissues from 5-week-old plants were used for the analysis. The relative expression of *PtS1Fa1* and *PtS1Fa2* in the mature leaves was set as 1 to normalize its expression in other tissues. **(B)** Expression profiles of *PtS1Fa1* and *PtS1Fa2* in young leaves of *P. trichocarpa* responding to 150 mM NaCl. **(C–F)** Young leaves were used for analysis under the conditions of 20% PEG6000 treatment **(C)**, 100 μM Me-JA treatment **(D)**, 100 μM SA treatment **(E)**, and 200 μM ABA treatment **(F)**. All the solutions were watered on roots of *P. trichocarpa* and *P. trichocarpa* plants growing under normal conditions were served as the mock controls. The expression values were converted by log2. Error bar indicates the standard deviation (STD) of the three biological replicates. Asterisk (^∗^) represents significant difference compared with WT (*P* < 0.05).

We use real-time quantitative PCR to verify the expression of the S1Fa genes in different tissues. qRT-PCR showed that S1Fa was expressed in various organizations, but the S1Fa family genes are mainly expressed in young leaves, and have low expression in other studied tissues. The expression profiles of the genes for PtS1Fa1 and PtS1Fa2 were generally consistent (Figure 4A). It shows that S1Fa TFs expression has tissue-specific characteristics. PtS1Fa1 and PtS1Fa2 were highly inhibited by salt and PEG stress during the treatment. Under salt stress conditions, the expression levels of PtS1Fa1 and PtS1Fa2 were highly decreased after stress for 1 and 6 h. PEG was applied to simulate a drought stress. Under PEG induced stress conditions, the expression levels of both PtS1Fa1 and PtS1Fa2 kept decreasing from 1 to 9 h, but recovered under stress for 12 h (Figures 4B,C). The transcript level of *PtS1Fa* was decreased first and then was gradually increased from 3 to 12 h under MeJA conditions (Figure 4D). When exposed to ABA and SA, the expression levels of *PtS1Fa1* and *PtS1Fa2* also showed an expression trend that first decreased first, then increased and then decreased again. The expression level of *PtS1Fa2* reached the lowest after JA treatment for 3 h, but the expression of PtS1Fa1 reached peak at 9 h. When treated with SA, the expression of *PtS1Fa* reaching a decreased peak at 3 h (Figures 4E,F).

### PtS1Fa1 and PtS1Fa2 Proteins Were Localized to the Nucleus

**FIGURE 5:**
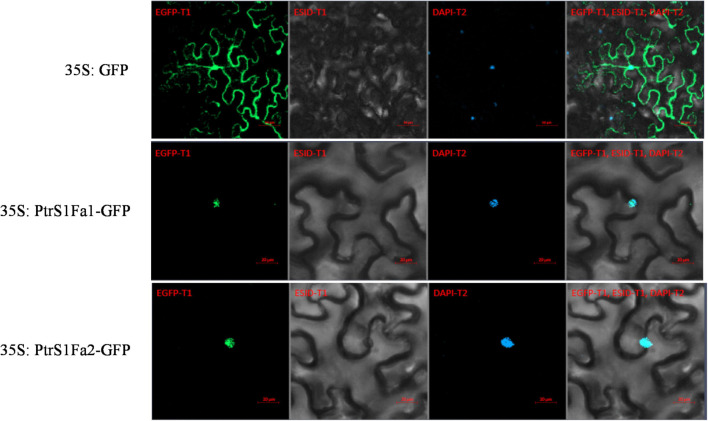
Cellular localization of the S1Fa-like protein. The constructs 35S:*S1Fa*1-GFP, 35S:*S1Fa*2-GFP, and 35S:GFP were, respectively, transformed into leaves of tobacco using Agroinfiltration; DAPI: staining of nuclei using DAPI; GFP: detection of GFP fluorescence; merge: the images of brightfield, DAPI staining, and GFP were merged together; ESID: Brightfield.

To analyze the subcellular location of PtS1Fa1 and PtS1Fa2, there was found that the 35S:S1Fa-GFP fusion protein could only observe green fluorescence in the nucleus in tobacco epidermal cells through confocal microscopy. And further we found that the signal overlapped staining with DAPI. By contrast, the GFP signal was evenly distributed in the tobacco plant cells transformed with 35S:GFP. These results indicated that both PtS1Fa1 and PtS1Fa2 are localized to the nucleus of plant cells (Figure 5).

### Generation of *Populus trichocarpa* Overexpressing PtS1Fa1 and PtS1Fa2

**FIGURE 6:**
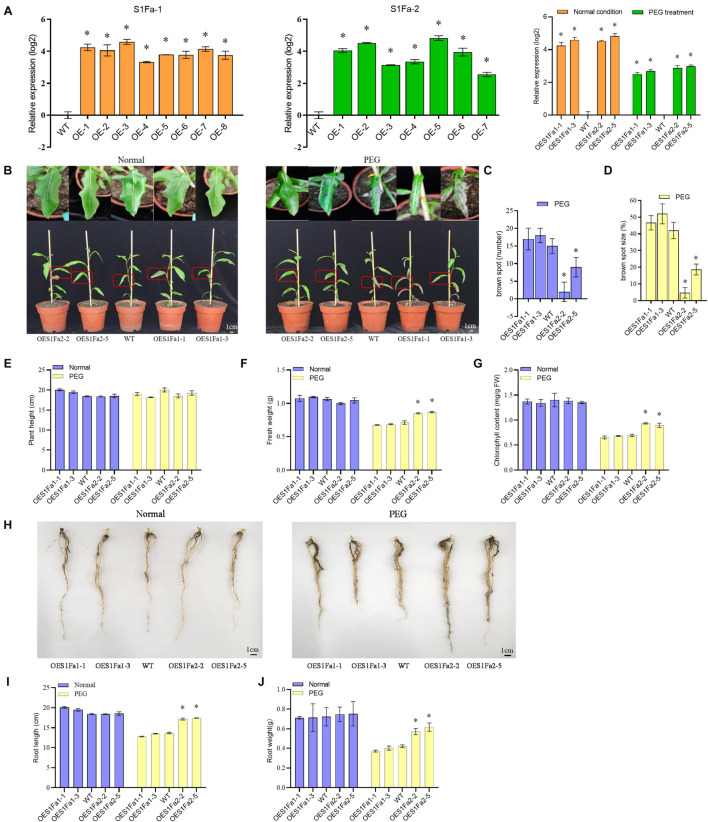
PtS1Fa2 confers drought tolerance. **(A)** Determination of the overexpression of *PtS1Fa1* and *PtS1Fa2* in transgenic lines (OE). The relative expression of *PtS1Fa1* and *PtS1Fa2* in OE lines were divided by that in WT plants, and the ratios were log2 transformed. **(B)** Comparison of the growth of OE lines and WT plants. **(C)** The number of brown spots. **(D)** The percentage of Brown spots size **(E–G)** Measurement of the plant height **(E)**, fresh weight **(F)**, and chlorophyll content **(G)**. **(H–J)** Comparison of the root phenotype **(H)**, root length **(I)**, and root weight **(J)** of OE and WT lines under normal conditions or the conditions of drought stress. Control: The well-watered plants were grown under normal conditions. All the plants were grown in the greenhouse at 23°C under a 16/8 h light/dark cycle and well-watered. PEG: 20% PEG6000 treatment for 15-day. The data represent means of three independent experiments. Each sample contains at least 3 plantlets. The error bar represents the standard deviation (STD) of the three biological repetition. Asterisks indicate significant differences between transgenic strains and WT (*P* > 0.05).

The transgenic *P. trichocarpa* plants overexpressing *PtS1Fa1* (termed OEPtS1Fa1) and *P. trichocarpa* plants overexpressing PtS1Fa2 (termed OEPtS1Fa2) were generated, respectively. The transcript level of P*trS1Fa1* and *PtrS1Fa2* in each transgenic line was determined using qRT-PCR. Compared with in WT plants, the expression of *PtrS1Fa1* and *PtrS1Fa2* was significantly elevated in the lines OEPtS1Fa1 or OEPtS1Fa2, respectively, (Figure 6A), indicating that *PtrS1Fa1* and *PtrS1Fa2* had been successfully transformed and expressed. We selected the lines of PtS1Fa1 (OE1-1 and 1-3) and PtS1Fa2 (OE2-2 and 2-5) with highest expression levels for further experiments. We further determined the selected lines and determined their expression under normal or stress conditions. The results showed that under PEG induced drought stress conditions, the expression of both PtS1Fa1 and PtS1Fa2 is decreased compared with under normal conditions (Figure 6A).

### Investigation of Drought Tolerance Mediated by PtS1Fa1 and PtS1Fa2

To examine whether *PtS1Fa1* and *PtS1Fa2* play roles in drought stress tolerance, we studied the drought tolerance of the OEPtS1Fa1, OEPtS1Fa2, and WT lines at the vegetative growth stage. The poplar plants were grown under normal or drought stress conditions, and their growth phenotypes were determined. Under normal growth conditions, all the studied plants displayed similar growth phenotype (Figure 6B). When exposed to drought conditions, no significant difference in plant heights was observed among OEPtS1Fa1, OEPtS1Fa2, and WT plants (Figures 6B,E). However, the leaves of the WT plants showed obvious drought damage, manifesting as brown spots. The leaves of OEPtS1Fa1-1 and OEPtS1Fa1-3 had 17 and 18 brown spots, and WT plants had 15 spots; however, OEPtS1Fa2-2 and OEPtS1Fa2-5 only had 2 and 9 spots (Figure 6C). In addition, the brown spots in OEPtS1Fa1-1 and OEPtS1Fa1-3 account for 46 and 52% total leave area, and in WT account for 42% total leave area. However, the brown spots in leaves of OEPtS1Fa2-2 and OEPtS1Fa2-5 only accounted for 4.7 and 18.7% of total area (Figure 6D). At the same time, the OEPtS1Fa2 lines had the significant higher fresh weights than both WT and OEPtS1Fa1 plants under drought stress (Figure 6F). In addition, under drought stress, OEPtS1Fa2 plants had significant higher chlorophyll contents than WT and OEPtS1Fa1 plants (Figure 6G). In addition, the OEPtS1Fa2 lines had both significant higher of root length and root weight than WT and OEPtS1Fa1 plants (Figures 6H–J), suggesting that OEPtrS1Fa2 can increase the drought resistance of plants by increasing root length and lateral root density, while, OEPtrS1Fa1 failed in conferring drought tolerance.

### Determination of Malondialdehyde, Reactive Oxygen Species Content, and Reactive Oxygen Species Scavenging Capability Mediated by PtS1Fa1 and PtS1Fa2

**FIGURE 7:**
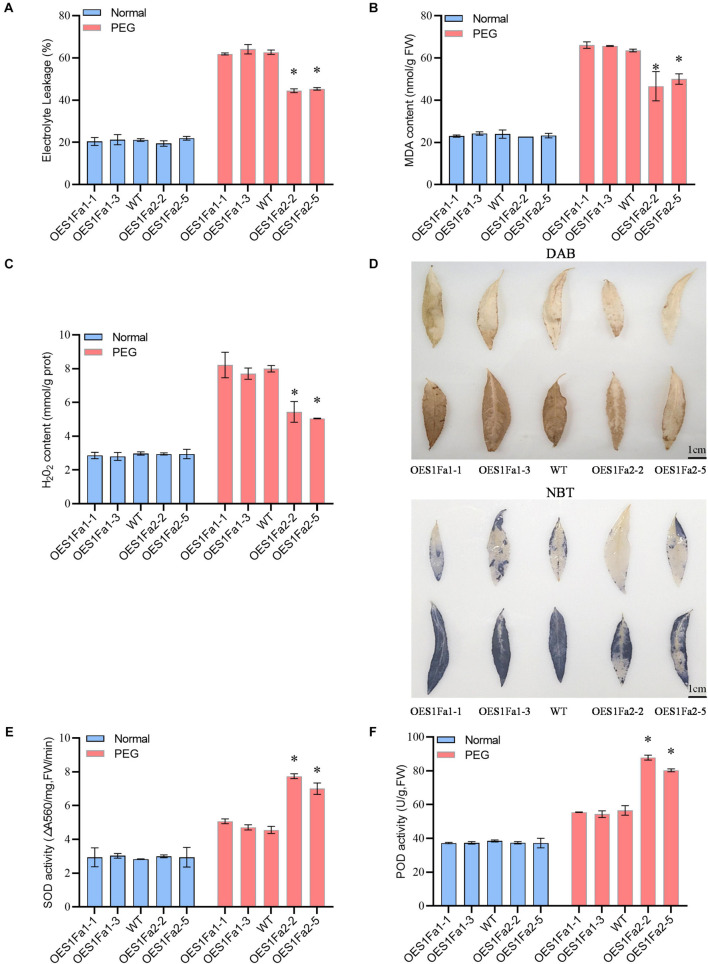
Analysis of ROS scavenging regulated by *PtS1Fa1* and *PtS1Fa2*. **(A)** Analysis of relative electrolyte leakage among WT, OEPtS1Fa1, and OEPtS1Fa2 plants. **(B)** MDA analysis of WT, OEPtS1Fa1, and OEPtS1Fa2 plants. (C) Analysis of H_2_O_2_ levels. **(D)** Detection of O_2_^–^ and H_2_O_2_^–^using NBT and DAB staining. The poplar plants were exposed to 20% PEG6000 for 0 (without stress) or 9 h, and their leaves were detached for NBT and DAB staining. **(E)** Measurement of SOD activity among WT, OEPtS1Fa1, and OEPtS1Fa2 plants. **(F)** Measurement of POD activity among WT, OEPtS1Fa1, and OEPtS1Fa2 plants. Each sample contains at least 3 plantlets. The error bar represents the standard deviation (STD) of the three biological repetition. Asterisks (^∗^) indicates significant differences between transgenic strains and WT (*P* > 0.05).

To determine the drought tolerance among the WT and OE lines. All the studied plants showed similar electrolyte leakage rates under normal conditions. However, under drought stress conditions, OEPtS1Fa1 plants has a similar electrolyte leakage rate to the WT poplar plants. However, compared with WT plants, OEPtS1Fa2 plants had significantly decreased electrolyte leakage (Figure 7A). OEPtS1Fa2 plants had a reduced MDA content compared with that in the WT plants did, whereas the MDA contents in the PtS1Fa1 and WT plants were similar. However, all the studied plants had similar MDA contents under normal growth conditions (Figure 7B). We further determined the H_2_O_2_ contents among the studied plants. The results showed that all the studied plants had similar H_2_O_2_ contents under normal conditions. Under drought conditions, OEPtS1Fa1 and WT plants had similar H_2_O_2_ content. By contrast, the OEPtS1Fa2 plants had significantly reduced H_2_O_2_ contents relative to the WT plants (Figure 7C). Consistently, both DAB (reflecting H_2_O_2_ contents) and NBT (O_2_^–^) staining showed that all these plants had similar H_2_O_2_ and O_2_^–^ contents under normal conditions. However, OEPtS1Fa2 plants displayed significantly lower level of H_2_O_2_ and O_2_^–^ contents relative to WT plants; however, the OEPtS1Fa1 plants showed similar H_2_O_2_ and O2- contents to those in the WT plants (Figure 7D). All the studied plants had similar SOD and POD activities under normal conditions. Under drought conditions, the SOD and POD activities in OEPtS1Fa2 plants were significantly higher than those in the WT plants, whereas those in the OEPtS2Fa1 plants and WT plants were similar (Figures 7E,F). Therefore, OEPtS1Fa*2* can decrease ROS accumulation, the MDA contents, and cell membrane damage.

**FIGURE 8:**
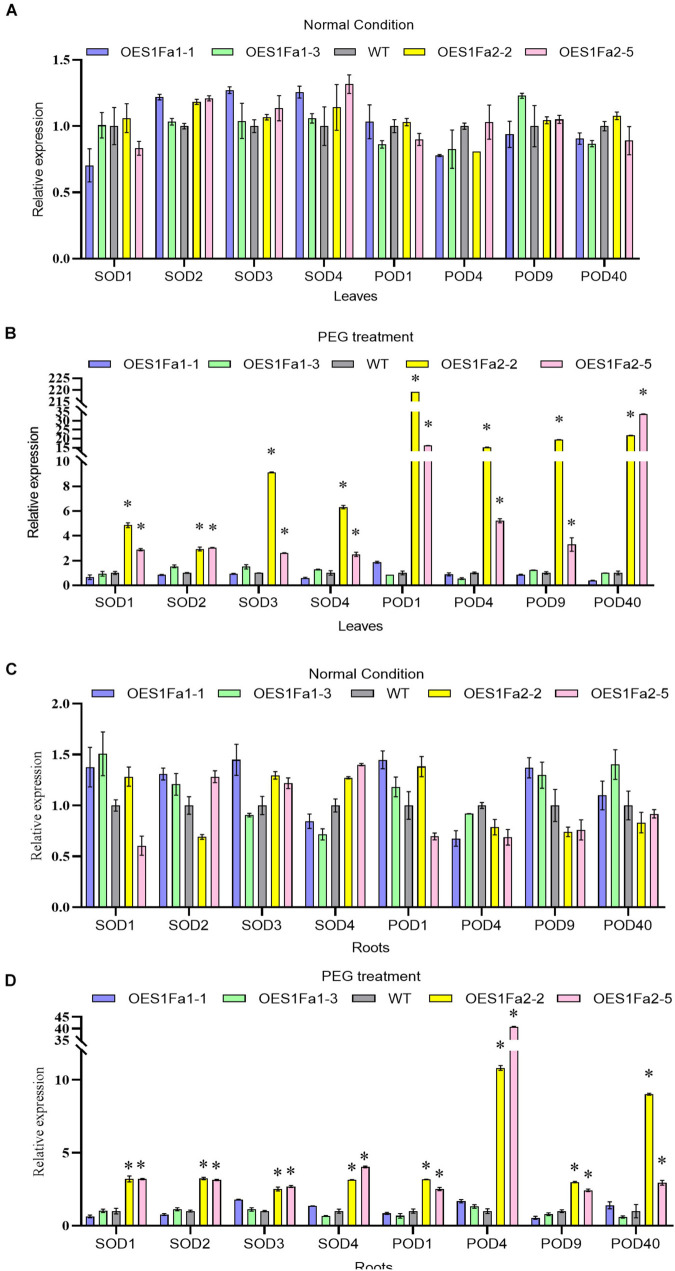
Determination of the expression of *POD* and *SOD* genes regulated by *PtS1Fa1* and *PtS1Fa2*. Analysis of the expression of *POD* and *SOD* genes in leaves of WT, *PtS1Fa1* and *PtS1Fa2* transformed lines (OEPtS1Fa1, and OEPtS1Fa2) using qRT-PCR under normal condition **(A)** or under PEG treatment conditions **(B)**. The expression of *POD* and *SOD* genes in roots of WT, OEPtS1Fa1, and OEPtS1Fa2 using qRT-PCR under normal condition **(C)** or under PEG treatment conditions **(D)**. Plants were treated with 20% (w/v) PEG6000 for 9 h, and samples were harvested for determination gene expression. The transcript level of genes in WT plants were used as control (set as 1) to normalize the gene expression in transgenic lines. Error bar represents the standard deviation (STD) of the three biological replicates. Asterisk (^∗^) indicates the significant differences at *P* < 0.05 compared with the WT.

As SOD and POD activities were altered by OEPtS1Fa2, we further studied whether *PtS1Fa1* and *PtS1Fa2* could regulate *SOD* and *POD* gene expression. All the studied plants displayed similar expression levels of PODs and SODs under normal conditions in roots and leaves (Figures 8A,C). However, under drought stress conditions, only *PtS1Fa2* could regulate the *SOD* and *POD* genes in both roots and leaves, whereas *PtS1Fa1* failed in regulation of the expression of genes for *POD* and *SOD* in roots and leaves (Figures 8B,D). These results suggested that PtS1Fa2 induces the expression of *SOD* and *POD* genes to increase SOD and POD activity, which can elevate the ROS scavenging capability to alleviate the damage caused by excess ROS under drought stress condition, leading to improved drought tolerance.

### Hypoxia Gene Expression Mediated by PtS1Fa1 and PtS1Fa2

**FIGURE 9:**
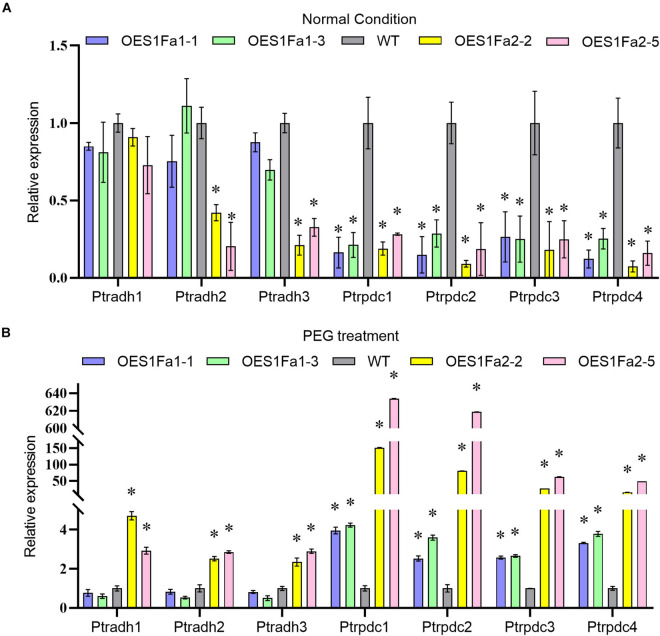
Analysis of the expression of hypoxia genes regulated by *PtS1Fa1* and *PtS1Fa2*. Analysis of hypoxia gene expression in OEPtS1Fa1, OEPtS1Fa2 and WT plants under normal **(A)** or PEG treatment conditions **(B)**. Plants are treated with 20% (w/v) PEG6000 for 9 h, and were used for qRT-PCR. Error bars represent the standard deviation (STD) of three biological replicates. The asterisk indicates significant difference between the genetically modified strain and WT (*P* > 0.05).

As drought stress will cause hypoxia, we studied the expression of hypoxia response genes, including three Alcohol dehydrogenase (*ADH*) genes and four Pyruvic dehydrogenase (*PDC*) genes. The expression of all studied *ADH* genes in the OEPtS1Fa1 were similar with WT poplar whether under drought or normal conditions. However, the expression of all studied *PDC* genes were significantly down-regulated under normal conditions, but were increased under drought conditions. All the studied *PDC* and *ADH* genes were down regulated in OEPtS1Fa2 plants under normal condition, but all of them were increased in OEPtS1Fa2 plants under drought stress conditions (Figure 9).

## Discussion

### PtS1Fa2 Confers Drought Tolerance

Although S1Fa-like TFs have been found to be involved in photomorphogenesis, their functions are still poorly studied (Gazara et al., 2019). Here, we cloned two genes of the S1Fa-like family, *PtS1Fa1* and *PtS1Fa2*, from *P. trichocarpa*. The analysis of protein motifs shows that motif1 is very conservative and contains major function (Figure 2A). The subcellular localization analysis of this study showed that two S1Fa-like TFs were localized in the nucleus, which was consistent with the reported localization. Our results showed that both *PtS1Fa1* and *PtS1F*a2 can be inhibited by drought and salinity stress, suggesting that they play roles in abiotic stress response (Figures 4B,C). After drought treatment, further studies showed that OEPtS2Fa2 displayed increased fresh weight, root length, and chlorophyll content compared with those in WT plants; thus, the growth phenotype of the OEPtS2Fa2 lines was better than WT plants, suggesting that OEPtS1Fa2 confers drought tolerance (Figure 6). However, OEPtS1Fa1 shared similar fresh weight, root length, chlorophyll content, and growth phenotype with WT plants, indicating that OEPtS1Fa1 failed to confer drought tolerance (Figure 6). Although *PtS1Fa1* and *PtS1Fa2* belong to the S1Fa-like TF family and share very similar expression profiles in responding to drought and salt stresses, they had different function in drought stress tolerance, indicating that S1Fa-like TF family members have diverse functions.

The promoter of *PtS1Fa1* and *PtS1Fa2* both contain stress related or hormone regulatory elements (Figure 3B), but they contain different motifs, indicating that *PtS1Fa1* and *PtS1Fa2* TFs are both involved in response to exogenous hormones and stress treatment, but may have different expression profiles. Consistently, the expression profiles of *PtS1Fa1* and *PtS1Fa2* was significantly different in response to MeJA, ABA, or SA treatment (Figures 4D–F), which are consistent with the analysis of the motifs in the promoters, also suggesting that *PtS1Fa1* and *PtS1Fa2* may have different function.

### Overexpression of PtS1Fa2 Improved the Reactive Oxygen Species Scavenging Capability to Enhance Drought Tolerance

As sessile organisms, plants have evolved extensive mechanisms to adapt and acclimate to counter the danger posed by adverse environment. ROS include free radicals such as hydroxyl radical (OH), non-radical molecules like hydrogen peroxide (H_2_O_2_), superoxide anion (O_2_^–^), as well as singlet oxygen (^1^O_2_). ROS have dual roles in plant responses to abiotic stress, functioning as important signal transduction molecules at low levels, as well as being toxic by-products of stress metabolism (Miller et al., 2010). When exposed to adverse environmental conditions, plants will generate excess ROS. A high level of ROS will be extremely harmful. When the ROS concentration exceeds the capacity of the defense mechanisms of cells, secondary stress, termed “oxidative stress” will occur. Excess ROS will cause oxidation of proteins, enzyme inhibition, peroxidation of lipids, damage to nucleic acids, and activation of programmed cell death (PCD) pathways; these changes ultimately lead to the death of the cells (Mittler, 2002; Meriga et al., 2004; Zhao et al., 2021). Therefore, maintaining ROS at a proper level is necessary for plants to adapt adverse environments. SOD, POD and catalase (CAT) are the defense enzymes that can eliminate ROS effectively to maintain the redox balance. In the present study, our results showed that OEPtS1Fa2significantly reduced H_2_O_2_ and O_2_^–^ levels. At the same time, OEPtS1Fa2 lines showed increased activities of SOD and POD compared with WT plants. Moreover, the expression of *POD* and S*OD* genes were all induced in OEPtS1Fa2 lines. We had screened the promoters of *SOD* genes, and no S1Fa binding sites were identified, suggesting that S1Fa can not directly regulate the expression of these SODs. Therefore, it should be that S1Fa regulates the TFs that containing the S1Fa binding sites in their promoters, and these TFs then regulate the expression of *SOD* genes. These results together indicated that PtS1Fa2 can induces the expression of *POD* and *SOD* genes, giving rise to increase POD and SOD activity. Finally, the increased activities of SOD and POD would reduce ROS accumulation, leading to improved drought tolerance.

### The Increase in Root Growth Might Contribute to Improved Drought Tolerance

Roots are the most important organ for plants to adapt salt or drought stress. The yield of dryland crops is strongly affected by the ability of plant roots to absorb stored moisture from deep soil (Wasson et al., 2012). The length of plant roots is a key morphological indicator of drought tolerance (O’Toole and Soemartono, 1981). Previous studies also indicated that plant varieties with good root growth usually possess higher drought tolerance (Reicosky and Deaton, 1979). For instance, overexpression of *OsNAC5* enlarged the diameter of rice root, leading to increased drought tolerance (Jeong et al., 2013). Overexpression of *TaRNAC1*, a predominantly root-expressed TF, can increase wheat biomass, root length, and drought tolerance (Chen et al., 2018). In this study, OEPtS1Fa2 increased the root weight and root length significantly compared with WT poplar plants (Figures 6F,H). Correspondingly, OEPtS1Fa2 shows the ability of drought tolerance remarkably, suggesting that the longer roots might increase the capability of the OEPtS1Fa2 root system to extract stored soil moisture, leading to improved drought tolerance (Figure 6).

### PtS1Fa1 and PtS1Fa2 Induce the Expression of Hypoxia-Responsive Genes

As previous studies showed that drought can induce hypoxia stress in plants (Manter and Kelsey, 2008), therefore, we further determined the expression of the genes related with hypoxia. After receiving the hypoxia signal, plants usually induce a series of gene expression to reduce damage caused by hypoxia, such as *ADH GAPC* and *PDC* genes. These genes are expressed lower under normal conditions, but the translation activity is greatly enhanced to adapt to low oxygen environment under hypoxia conditions (Sheila et al., 2002). In this study, under drought conditions, OEPtS1Fa2 can significantly increase the expression of ADH and PDC (Figure 9), suggesting that it can improve drought stress by induction of the expression of *ADH* and *PDC* to reduce damage caused by hypoxia. However, OEPtS1Fa1 can’t induce the expression of the studied *ADH* genes (Figure 9), which may be one of the reasons that it can’t confer drought tolerance.

### The Putative Target Genes Regulated by S1Fa-Like Transcription Factors

To analysis of the putative target genes of *PtS1Fa2*, we screened the S1F binding sites (“ATGGTAACAATT”) in the promoter of genes on the genomes of Populus (Supplementary Figure 1A). The putative target genes were further analyzed using GO annotations. The results showed that the putative target genes are involved in many pathways (Supplementary Table 2), such as regulation of gene expression, response to abiotic stress, protein binding, suggesting that *PtS1Fa2* were involved in these pathways. qRT-PCR was further performed to verify whether these genes are really regulated by *PtS1Fa1* and *PtS1Fa2*, and the result showed that most of these putative target genes were regulated by *PtS1Fa1* and *PtS1Fa2*, suggesting that the prediction is reliable. In addition, qPCR showed that *PtS1Fa2* regulates more putative target genes than genes are regulated by *PtS1Fa1* (Supplementary Figures 1B,C), which may explain the fact that *PtS1Fa2* could confer drought tolerance rather than *PtS1Fa1*. For instance, the expression of Potri.005G124000 (heme binding Protein) can be induced by *PtS1Fa2* but not be induced by *PtS1Fa1* (Supplementary Figure 1B). Previous studies showed that *Arabidopsis* Heme Binding Membrane Protein (TSPO) plays a role in porphyrin binding and scavenging during stress in plants (Vanhee et al., 2011). Therefore, the induction of Potri.005G124000 mediated by *PtS1Fa2* might be contributed to the adaption of drought stress.

## Data Availability Statement

The original contributions presented in the study are included in the article/Supplementary Material, further inquiries can be directed to the corresponding author/s.

## Author Contributions

YW directed and conceived the project. YN and HZ performed the experiments. HD and YJ performed the overall data analysis. HZ and YW wrote the manuscript. All authors contributed to the article and approved the submitted version.

## Conflict of Interest

The authors declare that the research was conducted in the absence of any commercial or financial relationships that could be construed as a potential conflict of interest.

## Publisher’s Note

All claims expressed in this article are solely those of the authors and do not necessarily represent those of their affiliated organizations, or those of the publisher, the editors and the reviewers. Any product that may be evaluated in this article, or claim that may be made by its manufacturer, is not guaranteed or endorsed by the publisher.

## Footnotes

1
http://meme-suite.org/

2
https://www.dna.affrc.go.jp/PLACE/?action=newplace
