# Supplementary material for: Characterization of the Function of Two S1Fa-Like Family Genes From Populus trichocarpa
Source: Front Plant Sci. 2021 Oct 4;12:753099. doi: 10.3389/fpls.2021.753099 (PMC8521066; doi:10.3389/fpls.2021.753099)
Supplement: Supplementary file 1 [file Data_Sheet_1.PDF]

Supplementary Figure1:

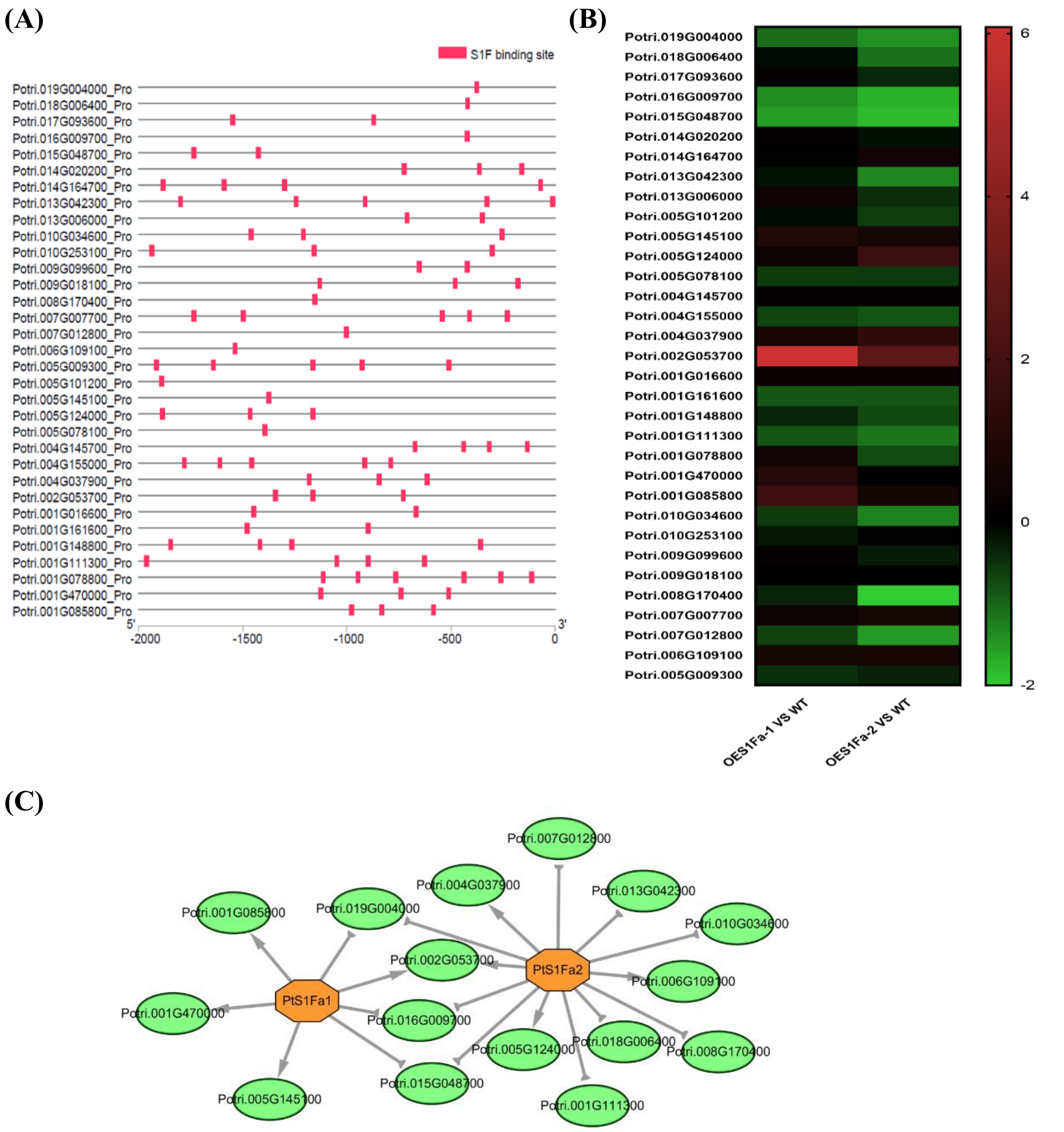

Supplementary Figure1: Analysis of S1Fa TFs binding S1F element regulatory genes expression

(A): Analysis of the gene promoters containing the cis-element binding site of S1F in *Populus trichocarpa*. **(B)**: qRT-PCR analysis of the expression of 33 genes in WT, OEPtS1Fa1 and OEPtS1Fa2 plants under drought stress conditions. There were 3 biological repeats for each sample. **(C):** Gene expression network of *PtS1Fa1* and *PtS1Fa2*. Orange hexagons indicate *PtS1Fa1* or *PtS1Fa2*; Green ellipses indicates the genes of *Populus trichocarpa*. Nodes in the graph represent different genes, and connections between nodes represent the regulatory relationship. Sharp arrows indicate induction of expression, flat and blunt arrows indicate expression inhibition.
