# Supplementary material for: Characterization of the Function of Two S1Fa-Like Family Genes From Populus trichocarpa
Source: Front Plant Sci. 2021 Oct 4;12:753099. doi: 10.3389/fpls.2021.753099 (PMC8521066; doi:10.3389/fpls.2021.753099)
Supplement: Supplementary file 3 [file Data_Sheet_2.PDF]

TableS2 *List of genes containing S1F cis-acting elements in the promoters of genes in Populus trichocarpa*

Cis-element	Target Gene ID	Name	GO Biological Process(**BP**) GO Cellular Component(**CC**) GO Molecular Function(**MF**)
S1F	Potri.019G004000	MNU	**BP** regulation of gene expression<br>**CC** chloroplast, mitochondrion, peroxisome<br>**MF** flowering stage, mature plant embryo stage, petal differentiation and expansion stage, plant embryo bilateral stage, plant embryo cotyledonary stage, plant embryo globular stage
	Potri.018G006400		**CC** nucleus
	Potri.017G093600		**CC** nucleus<br>**MF** RNA binding
	Potri.016G009700	SCL	**BP** regulation of transcription, DNA-templated transcription, DNA-templated<br>**CC** nucleus<br>**MF** DNA-binding transcription factor activity transcription regulatory region sequence-specific DNA binding
	Potri.015G048700		**CC** cytosol<br>**MF** carboxylic ester hydrolase activity, catalytic activity, acting on a tRNA
	Potri.014G020200		
	Potri.014G164700	HK	**BP** cellular response to abscisic acid stimulus, cellular response to cold, negative regulation of iron ion transport, phloem or xylem histogenesis, phosphorelay signal transduction system, regulation of flower development, regulation of meristem development, response to toxic substance, secondary growth<br>**CC** plasma membrane<br>**MF** cytokinin receptor activity, identical protein binding, kinase binding, osmosensor activity, phosphorelay sensor kinase activity, protein binding, protein histidine kinase activity, protein histidine kinase binding
	Potri.013G042300		
	Potri.013G006000		
	Potri.010G034600	PTST	**BP** starch biosynthetic process<br>**CC** chloroplast<br>**MF** maltoheptaose binding, polysaccharide binding, protein binding

	Potri.010G253100		**BP** defense response to fungus, regulation of flower development<br>**CC** nucleus
	Potri.009G099600		**BP** chloroplast proton-transporting ATP synthase complex assembly<br>**CC** chloroplast, chloroplast stroma<br>**MF** protein binding
	Potri.009G018100		**BP** heat acclimation, mRNA catabolic process<br>**CC** P-body, cytoplasm, cytoplasmic stress granule, cytosol, plastid<br>**MF** mRNA binding, protein binding
	Potri.008G170400		**BP** regulation of response to salt stress, regulation of response to water deprivation, vegetative to reproductive phase transition of meristem<br>**CC** cytoplasm, cytosol, nucleus<br>**MF** mRNA binding
S1F	Potri.007G007700		**CC** cytoplasm<br>**MF** ribosome binding
	Potri.007G012800		**BP** auxin-responsive<br>**CC** mitochondrion
	Potri.006G109100	WRKY	**BP** defense response to bacterium, defense response to fungus, defense response to oomycetes, floral organ senescence, induced systemic resistance, leaf senescence, positive regulation of salicylic acid mediated signaling pathway, regulation of brassinosteroid mediated signaling pathway, regulation of defense response, regulation of ethylene-activated signaling pathway, regulation of jasmonic acid mediated signaling pathway, regulation of response to osmotic stress, regulation of response to water deprivation, regulation of salicylic acid mediated signaling pathway, regulation of stomatal closure, regulation of transcription, DNA-templated, response to bacterium, response to insect, response to jasmonic acid, response to osmotic stress, response to reactive oxygen species, response to salicylic acid<br>**CC** nucleus<br>**MF** DNA-binding transcription factor activity, protein binding, sequence-specific DNA binding, transcription cis-regulatory region binding

	Potri.005G009300	RLP	**CC** extracellular region
	Potri.005G101200		**CC** nucleus
	Potri.005G145100		
	Potri.005G124000	CYP90B1	**BP** brassinosteroid biosynthetic process, brassinosteroid homeostasis, multicellular organism development, sterol metabolic process<br>**CC** endoplasmic reticulum<br>**MF** heme binding, iron ion binding, monooxygenase activity, oxidoreductase activity, oxidoreductase activity, acting on paired donors, with incorporation or reduction of molecular oxygen, steroid 22-alpha hydroxylase activity
	Potri.005G078100		**BP** glycolytic process<br>**CC** chloroplast
	Potri.004G145700		**BP** arginine biosynthetic process<br>**CC** chloroplast, chloroplast stroma, nucleolus<br>**MF** N-acetyl-gamma-glutamyl-phosphate reductase activity, NAD binding, copper ion binding, protein dimerization activity
S1F	Potri.004G155000		**BP** phenylalanyl-tRNA aminoacylation<br>**CC** cytoplasm, plasma membrane<br>**MF** phenylalanine-tRNA ligase activity, tRNA binding
	Potri.004G037900	TPS	**BP** diterpenoid biosynthetic process, response to bacterium, response to herbivore<br>**CC** chloroplast<br>**MF** (E,E)-geranyllinalool synthase activity, magnesium ion binding, terpene synthase activity
	Potri.002G053700		
	Potri.001G016600		**CC** nucleus<br>**MF** protein binding
	Potri.001G161600	SRF	**BP** protein phosphorylation<br>**CC** nucleus<br>**MF** protein binding, protein kinase activit
	Potri.001G148800		**BP** protein retention in Golgi apparatus, protein targeting to vacuole<br>**CC** cytosol, vacuole, plasma membrane, plasmodesma

S1F	Potri.001G111300	NRP D	**BP** transcription, DNA-templated<br>**CC** nucleoplasm, nucleus<br>**MF** DNA-directed 5'-3' RNA polymerase activity
	Potri.001G078800	MLO	**CC** chloroplast
	Potri.001G470000		**BP** amino acid transport<br>**CC** membrane, plasma membrane<br>**MF** amino acid transmembrane transporter activity
	Potri.001G085800		**CC** chloroplast
